# Using published data in Mendelian randomization: a blueprint for efficient identification of causal risk factors

**DOI:** 10.1007/s10654-015-0011-z

**Published:** 2015-03-15

**Authors:** Stephen Burgess, Robert A. Scott, Nicholas J. Timpson, George Davey Smith, Simon G. Thompson, EPIC- InterAct Consortium

**Affiliations:** 1 Department of Public Health and Primary Care, University of Cambridge, Cambridge, UK; 2 MRC Epidemiology Unit, University of Cambridge, Cambridge, UK; 3 MRC Integrative Epidemiology Unit, University of Bristol, Bristol, UK

**Keywords:** Mendelian randomization, Instrumental variable, Causal inference, Published data, Two-sample Mendelian randomization, Summarized data

## Abstract

Finding individual-level data for adequately-powered Mendelian randomization analyses may be problematic. As publicly-available summarized data on genetic associations with disease outcomes from large consortia are becoming more abundant, use of published data is an attractive analysis strategy for obtaining precise estimates of the causal effects of risk factors on outcomes. We detail the necessary steps for conducting Mendelian randomization investigations using published data, and present novel statistical methods for combining data on the associations of multiple (correlated or uncorrelated) genetic variants with the risk factor and outcome into a single causal effect estimate. A two-sample analysis strategy may be employed, in which evidence on the gene-risk factor and gene-outcome associations are taken from different data sources. These approaches allow the efficient identification of risk factors that are suitable targets for clinical intervention from published data, although the ability to assess the assumptions necessary for causal inference is diminished. Methods and guidance are illustrated using the example of the causal effect of serum calcium levels on fasting glucose concentrations. The estimated causal effect of a 1 standard deviation (0.13 mmol/L) increase in calcium levels on fasting glucose (mM) using a single lead variant from the *CASR* gene region is 0.044 (95 % credible interval −0.002, 0.100). In contrast, using our method to account for the correlation between variants, the corresponding estimate using 17 genetic variants is 0.022 (95 % credible interval 0.009, 0.035), a more clearly positive causal effect.

**Electronic supplementary material:**

The online version of this article (doi:10.1007/s10654-015-0011-z) contains supplementary material, which is available to authorized users.

## Introduction

Mendelian randomization is a technique which uses genetic variants to assess whether a risk factor, such as a biomarker, has a causal effect on a disease outcome in a non-experimental (observational) setting [1, 2]. We assume that the chosen genetic variants are associated with the risk factor, but not associated with any confounder of the risk factor–outcome relationship, nor associated with the outcome via any pathway other than that through the risk factor of interest [3]. These three assumptions form the definition of an instrumental variable [4]. A variant satisfying these assumptions divides a study population into subgroups which are analogous to treatment arms in a randomized controlled trial, in that they differ systematically with respect to the risk factor of interest, but not with respect to confounding factors [5]. An association between the genetic variant and the outcome therefore implies that the risk factor has a causal effect on the outcome.

Mendelian randomization is a valuable approach for identifying risk factors as potential targets for clinical or behavioural intervention [6]. Evidence from Mendelian randomization has been used to prioritize investigation of certain biomarkers as causal risk factors for cardiovascular disease: for example lipoprotein(a) [7], and interleukin-6 receptor [8]; and to de-prioritize others: fibrinogen [9], C-reactive protein (CRP) [10], and uric acid [11]. However, it may be hard to find a suitable study population with sufficient data on the genetic variants, and both the risk factor and outcome of interest. As many genetic variants only explain a small proportion of the variation in the risk factor, large sample sizes (in some cases comprising tens of thousands of individuals [12]) may be required for adequately-powered Mendelian randomization investigations. Several consortia with large numbers of participants, such as CARDIoGRAMplusC4D for coronary artery disease [13] and DIAGRAM for type 2 diabetes [14], have published data on the association of catalogues of genetic variants with either risk factors or disease status (a list of consortia is given in Web Table A1). These provide precise estimates of genetic associations which can be used to obtain causal estimates based on Mendelian randomization in a fast and cost-effective way. In this paper, we provide a blueprint for this approach.

## Methods

**Fig. 1:**
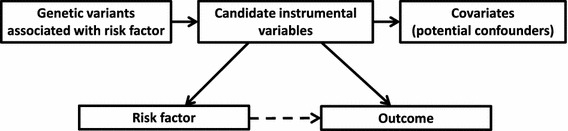
Schematic diagram outlining the Mendelian randomization approach

The steps involved in a Mendelian randomization investigation are: (1) specification of the dataset(s) for analysis, (2) search for candidate instrumental variables, (3) validation of the instrumental variable assumptions, (4) estimation of the causal effect (if appropriate), (5) supplementary and sensitivity analyses. A schematic diagram of the relevant components in a Mendelian randomization analysis is given in Fig. 1. We proceed to outline each of these steps.

### Specification of the dataset(s) for analysis

Traditionally, Mendelian randomization analyses have been performed on a single study or studies containing data on genetic variants, and both the risk factor and outcome of interest. The main advantages of using published data rather than individual-level data are their size and scope. The associations of these variants with the risk factor and outcome in large consortia are likely to be more precisely estimated than in a single study. However, it is unlikely that published data on the genetic associations with the risk factor, with the outcome, and with potential confounders are available on the same set of studies.

Two-sample Mendelian randomization is a strategy in which evidence on the associations of genetic variants with the risk factor and with the outcome comes from non-overlapping data sources [15]. The limiting factor for the power of a Mendelian randomization analysis using a given set of genetic variants is the precision in the estimate of the genetic association with the outcome, as this association is typically much weaker than the genetic association with the risk factor. Published data on genetic associations with the outcome can therefore be combined with individual-level data from a cross-sectional study on genetic variants and the risk factor to obtain precise Mendelian randomization estimates. If the study used to estimate genetic associations with the risk factor is included in the estimate of the genetic association with the outcome, then this is a subsample rather than a two-sample analysis strategy. Alternatively, published data can be used in all aspects of the analysis. In this case, the two published data sources may overlap (for example, they both constitute meta-analyses and some studies are included in both sources).

In any case, it is likely that the sets of individuals used in the gene-risk factor and gene-outcome arms of the analysis will not be identical. An important assumption to ensure the validity of the analysis is that the two sets represent samples taken from the same underlying population. If this is not the case, then inferences may be misleading, as the association of the genetic variants with the risk factor may not be replicated in the set of individuals in which the association with the outcome is estimated, or a variant may not be a valid instrumental variable in both sets.

### Search for candidate instrumental variables

Genetic variants are sought which are associated with the risk factor of interest. These can be obtained from available individual-level data or from the catalogues of genetic variants identified by genome-wide association studies (GWAS) that have been compiled [16]. It is important that estimates of both the gene-risk factor and the gene-outcome associations are available for each of these variants, or for proxies of the variants (a proxy is a variant in complete or near complete linkage disequilibrium with the original variant).

In two-sample Mendelian randomization, any bias from weak instruments (instrumental variables that are not strongly associated with the risk factor) is in the direction of the null [17], so the use of large numbers of genetic variants which are valid instrumental variables should not result in causal claims which are false positives. If the same set of individuals is used for estimating both the gene-risk factor and gene-outcome associations, then bias of the causal effect estimate will be in the direction of the observational association between the risk factor and the outcome. In subsample Mendelian randomization, or if the data sources for the associations overlap, the net bias will depend on the degree of overlap. If the overlap is not substantial, then it should be in the direction of the null [15].

### Validation of the instrumental variable assumptions

The instrumental variable assumptions for a genetic variant, or set of variants, are vitally important to the validity of any Mendelian randomization investigation. However, the assumptions are not all empirically testable. This means that, while the assumptions should be interrogated as far as possible, they cannot be entirely verified and must be justified as much by biological understanding as they are by statistical testing.

The assumptions necessary for a genetic variant to be a valid instrumental variable are:

1. the variant must be associated with the risk factor of interest;
2. the variant must be independent of confounders of the risk factor–outcome association;
3. the variant can only affect the outcome through the risk factor—if the value of the genetic variant changes, but not that of the risk factor, then the outcome is unchanged [18].

With regard to biological understanding, if the function of the gene in which the variant is located is known, this may give a clue as to whether the variant is a plausible instrumental variable. For example, variants in the *CRP* gene are likely to be valid instrumental variables for CRP. However, few genetic variants discovered in GWAS investigations are located within coding regions or have functional follow-up ascribing their association to a particular gene, and so the functional relationship between a variant and the risk factor may not be clear.

With regard to statistical testing, the simplest and perhaps most effective way of assessing the instrumental variable assumptions is to test the association of the candidate genetic variants with a range of covariates which are potential confounders using individual-level data. While there is no way of testing the association of the variants with unknown or unmeasured confounders, for several diseases many of the covariates having the strongest association with the outcome (and therefore the greatest potential to bias causal effect estimates) are known and often measured in epidemiological studies. Associations with several covariates can also be assessed from the literature, for example by searching for associations of the variants in a GWAS catalogue [16]. However, a key advantage of individual-level data over published data for validation is the ability to test the associations of the candidate instrumental variables with a range of covariates in a systematic way.

One difficulty with this assessment of the instrumental variable assumptions is the problem of multiple testing. If there are many covariates and multiple genetic variants, then a hypothesis testing approach that accounts for the multiple comparisons may lead to a lack of power to detect any specific association. Additionally, as several covariates (or the genetic variants) may be correlated, a simple Bonferroni correction may be an over-correction. A second difficulty is that genetic variants can be associated with a covariate without violating the instrumental variable assumptions. If, for example, a genetic variant which is a candidate instrumental variable for body mass index (BMI) is also associated with blood pressure levels, this may be due to the causal effect of BMI on blood pressure and not due to a pleiotropic effect of the variant (pleiotropy means that a variant has multiple effects). If the genetic association with a covariate is entirely mediated through the risk factor of interest, then the instrumental variable assumptions are not violated. In this case, taking the example above, the coefficient in the regression of blood pressure on the genetic variant should be substantially attenuated on adjustment for BMI. However, attenuation may not be complete, due to possible measurement error in the intermediate variable (here, BMI), and as the genetic variant is not independent of blood pressure conditional on BMI due to the presence of confounding factors between BMI and blood pressure [3].

A practical way to proceed is to specify two sets of genetic variants to be used as instrumental variables: a ‘conservative’ set, for which the minimum *p* value for the association of each variant with a covariate is greater than a pre-specified level (say *p* > 0.01), and a ‘liberal’ set, for which the minimum *p* value for each variant is greater than the Bonferroni corrected *p* value ($p>\frac{0.05}{V}$ where *V* is the number of covariates tested). If this approach is followed, to minimize the possibility of bias due to pleiotropy, the Mendelian randomization estimate using the ‘conservative’ set of variants should be regarded as the primary analysis and the estimate using the ‘liberal’ set as the secondary analysis.

Other violations of the instrumental variable assumptions, such as population stratification, are more difficult to test using only summarized data. This particular issue is discussed in the Web Appendix in the context of the applied example.

### Estimation of the causal effect

We assume that estimates and standard errors (or equivalently estimates and *p* values) are available for the genetic associations with the risk factor and with the outcome. Initially we assume that the scenario is two-sample Mendelian randomization and all the genetic variants considered are uncorrelated (in linkage equilibrium). These assumptions are later relaxed.

#### Genetic variants uncorrelated (linkage equilibrium)

For each of *K* genetic variants ($k = 1, \ldots , K$), we represent the estimate of the genetic association with the risk factor as $X_k$ with standard error $\sigma _{Xk}$, and the estimate of the genetic association with the outcome as $Y_k$ with standard error $\sigma _{Yk}$. Usually, these genetic associations are per allele effects: the change in the risk factor or outcome for each additional copy of the minor (or effect) allele. If the outcome is binary, then $Y_k$ is usually the regression coefficient from a logistic regression, representing a log odds ratio.

Two methods have been proposed for the estimation of a causal effect from these summarized estimates: an inverse-variance weighted method [19], and a likelihood-based method [20]. When the genetic associations with the risk factor are precisely estimated, both approaches give similar estimates. When there is considerable imprecision in the estimates, causal effect estimates from the inverse-variance weighted method are over-precise, while the likelihood-based method gives appropriately-sized confidence intervals.

The causal estimate from the inverse-variance weighted method ($\hat{\beta }_{\mathrm{IVW}}$) is:

1 $$\begin{aligned} \hat{\beta }_{\mathrm{IVW}} = \frac{\sum _{k=1}^K X_k Y_k \sigma _{Yk}^{-2}}{\sum _{k=1}^K X_k^{2} \sigma _{Yk}^{-2}}. \end{aligned}$$

The approximate standard error of the estimate is:

2 $$\begin{aligned} {{\mathrm{se}}}(\hat{\beta }_{\mathrm{IVW}}) = \sqrt{\frac{1}{\sum _{k=1}^K X_k^{2} \sigma _{Yk}^{-2}}}. \end{aligned}$$

The inverse-variance weighted estimator can be motivated as a weighted average of the ratio estimates $\frac{Y_k}{X_k}$ for each variant $k$, weighted using the reciprocal of an approximate expression for their asymptotic variance $\frac{\sigma _{Yk}^2}{X_k^2}$ (inverse-variance weighting, as in a meta-analysis) [21]. The estimate $\hat{\beta }_{\mathrm{IVW}}$ expresses the causal increase in the outcome (or log odds of the outcome for a binary outcome) per unit change in the risk factor. The relationship between the risk factor and the outcome is assumed to be linear.

The estimate from the likelihood-based method ($\hat{\beta }_{L}$) is obtained from the likelihood function of the model:

3 $$\begin{aligned} X_k&\sim \mathcal {N}(\xi _k, \sigma _{Xk}^2) \\ Y_k&\sim \mathcal {N}(\beta _{L} \xi _k, \sigma _{Yk}^2) \text{ for } k = 1, \ldots , K. \nonumber \end{aligned}$$

Estimates and confidence intervals can be obtained by direct maximization of the likelihood, or from Bayesian methods. The likelihood-based method can be motivated as finding the linear relationship between the coefficients $X_k$ and $Y_k$ which best fits the data, allowing for the uncertainty in both sets of coefficients. As above, the likelihood-based estimator expresses the causal increase in the outcome per unit change in the risk factor assuming a linear association between the risk factor and outcome variables.

These models assume that the data sources for the association estimates with the risk factor and with the outcome are non-overlapping. If they overlap, then the coefficients $X_k$ and $Y_k$ will be correlated in their distributions. The likelihood-based method can be modified to accommodate this by considering a bivariate model of $(X_k, Y_k)$ for each genetic variant (see [20]).

#### Genetic variants correlated (linkage disequilibrium)

If the genetic variants are correlated, then estimates from the inverse-variance weighted method will overstate precision. If estimates are available of the correlations between variants, then the likelihood-based method can be modified by assuming a multivariate normal distribution for the genetic associations with the risk factor $\mathbf {X} = (X_k; k = 1, \ldots K)$ and with the outcome $\mathbf {Y} = (Y_k; k = 1, \ldots K)$, with estimates of these correlations used in the variance–covariance matrices. The correlation between the coefficients for the associations of two genetic variants with the risk factor (as well as with the outcome) are equal to the correlation between the variants themselves:

4 $$\begin{aligned} \mathbf {X}&\sim \mathcal {N}_K({\varvec{\xi }}, \varSigma _{X}) \\ \mathbf {Y}&\sim \mathcal {N}_K(\beta _{L} {\varvec{\xi }}, \varSigma _{Y}) \nonumber \end{aligned}$$

where the matrix component $\varSigma _{Xij} = \sigma _{Xi} \sigma _{Xj} \rho _{ij}$, with $\sigma _{Xi}$ being the standard error of the coefficient $X_i$ and $\rho _{ij}$ the correlation between variants $i$ and $j$ (and $\rho _{ii} = 1$ for all $i$). Likewise $\varSigma _{Yij} = \sigma _{Yi} \sigma _{Yj} \rho _{ij}$. Software code for implementing these methods is provided in the Web Appendix.

Again, if the data sources for the association estimates are overlapping then a joint normal model for the genetic associations $(\mathbf {X}, \mathbf {Y})$ can be estimated:

5 $$\begin{aligned} \left( \begin{array}{c} \mathbf {X} \\ \mathbf {Y} \\ \end{array} \right) \sim \mathcal {N}_{2K}\left( \left( \begin{array}{c} {\varvec{\xi }} \\ \beta _{L} {\varvec{\xi }} \\ \end{array} \right) , \left( \begin{array}{cc} \varSigma _{X} &{} \varSigma _{XY} \\ \varSigma _{YX} &{} \varSigma _{Y} \\ \end{array} \right) \right) \end{aligned}$$

where the matrix component $\varSigma _{XYij} = \theta \sigma _{Xi} \sigma _{Yj} \rho _{ij}$, with $\theta$ representing the correlation between the genetic associations with the risk factor and outcome, and $\varSigma _{XY} = \varSigma _{YX}^T$. The value of $\theta$ can be estimated by bootstrapping if the individual-level data is available; otherwise, sensitivity analyses can be undertaken across a range of plausible values.

### Supplementary and sensitivity analyses

In addition to the primary analysis to estimate the causal effect of the risk factor on the outcome, a number of additional analyses can be performed, which fall into the categories of supplementary or sensitivity analyses.

If there are multiple mechanisms by which the risk factor may affect the outcome, and if genetic variants can be categorized as relating to one or other of these mechanisms, then separate Mendelian randomization estimates can be obtained using each category of variants. For example, variants may be associated with BMI by various mechanisms, such as suppressing appetite or increasing metabolic rate. A Mendelian randomization estimate constructed using variants associated with BMI through appetite suppression more closely represents the causal effect of intervening on BMI via appetite suppression. Differences in the causal estimates using genetic variants associated with different mechanisms may be informative in understanding the aetiology of the disease, and may highlight specific mechanisms to prioritize for pharmacological intervention.

If there are variants whose status as instrumental variables is uncertain, then sensitivity analyses can be performed using a more conservative and a more liberal set of genetic variants, as described in step 3. Additionally, if there is no pleiotropy and the effects of the risk factor on the outcome associated with changes in the genetic variants are homogeneous for all variants, the genetic association estimates with the risk factor and with the outcome should follow a linear relationship passing through the origin. By plotting the genetic association estimates with the risk factor and with the outcome, any points which are not compatible with a straight-line through the origin (allowing for uncertainty in the estimates) can be investigated for potential pleiotropy of the variants or for heterogeneity of the causal effect (perhaps due to different mechanisms of association with the risk factor).

A formal test for heterogeneity is known as an overidentification test [22]. Examples of overidentification tests with individual-level data include the Basmann test [23] and the Sargan test [24]. A similar test can be derived with summarized data from the likelihood-based method to test the hypothesis that the causal effect $\beta _{L}$ is the same using all variants: if $\beta _{L}$ were replaced by $\beta _{Lk}$, are the differences between the $\hat{\beta }_{Lk}$ compatible with chance? By the likelihood ratio test, twice the difference in the log-likelihood function evaluated at the maximum likelihood estimate with $\beta _{Lk} = \beta _{L}$ and evaluated at $\xi _k = X_k$, $\beta _{Lk} \xi _k = Y_k$ (saturated model) should be distributed as a chi-squared variable on $K-1$ degrees of freedom under the null hypothesis of homogeneity.

## Example: effect of calcium levels on fasting glucose

Calcium is the most abundant mineral in the body, with a wide range of vital functions in human biology, including bone development and maintenance, muscle contraction, neurotransmitter release, and exocytosis. Indeed, insulin secretion is a calcium dependent process [25], and total serum calcium levels have been associated with glucose intolerance [26]. Calcium absorption is enhanced by vitamin D, and vitamin D is a putative causal risk factor for type 2 diabetes [27]. We perform a Mendelian randomization analysis to investigate the causal effect of serum calcium levels on fasting glucose concentrations to illustrate some of the points discussed above.

For the gene-risk factor associations, we use individual-level baseline data on 6351 subcohort participants of European ancestry from the EPIC-InterAct study, a multicentre case-cohort study of type 2 diabetes nested within the European Prospective Investigation into Cancer and Nutrition (EPIC) [28]. All participants gave written informed consent, and the study was approved by the local ethics committees in the participating countries and the Internal Review Board of the International Agency for Research on Cancer. For the gene-outcome associations, we use published data from the Meta-Analyses of Glucose and Insulin-related traits Consortium (MAGIC), downloaded from www.magicinvestigators.org [29]. Data on per allele genetic associations with fasting glucose are available for up to 133,010 participants without diabetes of European ancestry from 66 studies. EPIC-InterAct participants were not included in the MAGIC dataset, so this is a two-sample Mendelian randomization design. Genetic variants for both samples were available for variants on the Cardio-Metabochip (Illumina).

### Identification of candidate variants and assessment of instrumental variable assumptions

We compare two strategies for choosing genetic variants to include in the Mendelian randomization analysis. The first strategy is to include only variants from in and around the calcium-sensing receptor (*CASR*) gene region [30]. This region was shown to have the strongest association with calcium levels in a GWAS [31] and has known biological relevance for calcium metabolism pathways. There are 17 variants within a 500 kb range of the *CASR* gene in various degrees of linkage disequilibrium; the lead variant was rs1801725. The second strategy is to include ten variants from the different gene regions identified as associated with calcium levels by O’Seaghdha et al. [31]. Suitable proxies were found for the variants which are not available on the Cardio-Metabochip. Further details of the data and genetic variants used in the analysis are given in Web Tables A2 and A3.

**Fig. 2:**
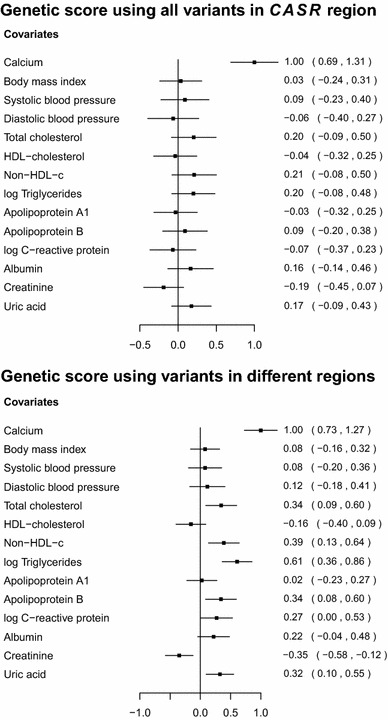
Associations with a range of covariates of weighted allele scores based on genetic variants associated with calcium levels for: (*top*) 17 variants in and around the *CASR* gene region; (*bottom*) 10 variants in different gene regions. Estimates are coefficients for the difference in the covariate measured in standard deviations per unit increase in the allele score [a unit increase in the allele score is scaled to be associated with a 1 standard deviation (0.13 mmol/L) increase in calcium levels]. Coefficients are obtained from the EPIC-InterAct dataset using linear regression with adjustment for age, sex and centre. Lines are 95 % confidence intervals

To assess the validity of the genetic variants as instrumental variables, we tested the association of the variants with a range of covariates in the EPIC-InterAct data. Associations of weighted allele scores based on the two sets of variants are displayed in Fig. 2. The weights for the allele scores were determined from the data under analysis by regression of calcium levels on each of variants in turn with adjustment for age, sex and centre. The regressions of the covariates on the allele scores were also adjusted for age, sex and centre. The use of weights derived from the data under analysis can lead to overfitting and weak instrument bias in a one-sample setting (genetic variants, risk factor and outcome measured in the same dataset), and so is not recommended for the primary Mendelian randomization analysis where it is important to mitigate against false positive results [32].

The coefficients represent the standard deviation difference in the covariate associated with a unit increase in the allele score [(which is scaled to be associated with a 1 standard deviation (0.13 mmol/L) increase in calcium levels]. The allele score based on variants from the *CASR* gene region does not show stronger associations with the covariates than would be expected by chance. A search of the literature revealed a suggestive association between cardiac troponin-T (a regulatory protein integral to muscle contraction) and a variant near to the *CASR* locus [33]. However, this association may be solely due to the genetic effect on calcium levels, in which case the Mendelian randomization assumptions are not violated. No other associations were reported. In contrast, the allele score based on variants from different gene regions is associated at $p<0.01$ with total cholesterol, non-high-density lipoprotein-cholesterol, triglycerides, apolipoprotein B, creatinine, and uric acid, and additionally at $p<0.05$ with CRP. Since summarizing a set of genetic variants as an allele score may hide pleiotropic effects of particular variants, associations of each of the variants individually with the covariates are given in Web Tables A4 and A5; this yields similar conclusions. A discussion on potential population stratification for variants in the *CASR* gene region is given in the Web Appendix.

### Estimation of a causal effect

We proceed to consider causal estimation only using the genetic variants in and around the *CASR* gene region. The restriction to a single genetic region means that the causal estimate is likely to apply only to a single mechanism by which calcium levels affect fasting glucose, and therefore may not be generalizable to other mechanisms. However, as the genetic region has a plausible mechanistic association with calcium levels, it is more likely to be a valid causal estimate than one based on variants from many genetic regions with unknown functional relevance to calcium levels and clear evidence of pleiotropy.

**Fig. 3:**
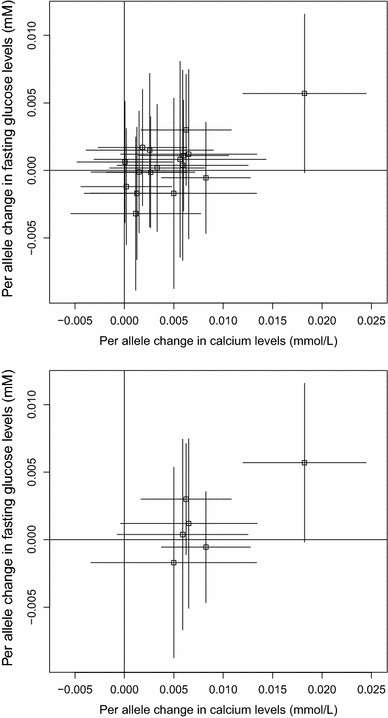
Association of genetic variants with fasting glucose (mM) obtained from publicly-available data from MAGIC consortium against association with calcium levels (mmol/L) obtained from EPIC-InterAct per calcium-increasing allele for: (*top*) 17 variants in and around the *CASR* gene region; (*bottom*) the subset of 6 variants in and around the *CASR* gene region associated with calcium levels ($p<0.1$). Lines represent 95 % confidence intervals

The genetic associations with calcium levels and with fasting glucose are displayed in Fig. 3. The top panel shows the associations for all 17 genetic variants, while the bottom panel only shows the associations for the 6 variants associated with calcium levels at $p<0.1$; this second analysis was conducted to mitigate the potential effects of weak instrument bias. However, the data-driven choice of instrumental variables can also lead to weak instrument bias [34]; hence the analysis using all variants regardless of their association with calcium levels is also performed.

Parameters in the likelihood based model (4) were estimated in a Bayesian framework; further details including the vague priors used are provided in the Web Appendix. The causal effect of calcium levels on fasting glucose is estimated using the full set of 17 variants, the subset of 6 variants, and the lead variant only (Table 1). The correlations between the genetic variants were estimated from the EPIC-InterAct data. The heterogeneity test statistics are: all variants 21.5 [16 degrees of freedom (*df*), $p=0.15$]; variants associated with calcium 3.28 (5 *df*, $p=0.66$), indicating no more heterogeneity in the genetic associations with the risk factor and outcome than would be expected by chance. The estimate using all the genetic variants is more precise than the estimate using only a subset of variants, even though the additional variants are not associated with calcium at nominally significant levels. This example shows the potential gain in power attained by using many genetic variants from a single gene region.

**Table 1:** Causal estimates for a 1 standard deviation (0.13 mmol/L) increase in calcium levels on fasting glucose (mM) using genetic variants from in and around the *CASR* gene region

	Number of variants	*F* statistic	Causal estimate	95 % credible interval
All variants	17	3.4	0.022	0.009, 0.035
Variants associated with calcium at *p* < 0.1	6	7.9	0.028	−0.003, 0.062
Lead variant only	1	30.6	0.044	−0.002, 0.100

Estimates and 95 % credible intervals are estimated from Bayesian likelihood-based method using all 17 measured variants, using the 6 variants associated with calcium in the EPIC-InterAct dataset (*p* < 0.1), and using the lead variant (rs1801725) only. Partial F statistics are taken from the regression of calcium on the genetic variants in a multivariable regression (with adjustment for age, sex, and centre)

We conclude from this example that there is evidence that increases in calcium levels lead to increases in fasting glucose. The lack of availability of data on important covariates (in particular vitamin D levels), the potential for bias by population stratification, and the reliance on genetic variants from a single region mean that the evidence that intervening to lower serum calcium levels would decrease fasting glucose concentrations is suggestive, but not conclusive.

## Discussion

In this discussion, we highlight some extensions of the approach discussed in this paper, as well as issues in its implementation and interpretation.

### Related risk factors and pleiotropic variants

In some cases, genetic variants are associated with several related risk factors, such as multiple lipid fractions (or several measures of the same risk factor, such as the concentration and particle size of lipoprotein(a)) in such a way that it is not possible to find variants specifically associated with each risk factor which are not associated with the related risk factors [35]. By considering the genetic associations with each of the risk factors in a single model, the causal effects of each of the risk factors on the outcome can be estimated simultaneously even from published data [36]. Such an analysis should only be attempted if the risk factors are closely biologically related and is only valid if the pleiotropic effects of the genetic variants are restricted to the set of risk factors under analysis.

### Multiple studies and meta-analysis

If the data on the genetic associations in a Mendelian randomization investigation are taken from multiple studies, then the association estimates may represent pooled estimates from a meta-analysis, as with the data on gene-outcome associations in the example of this paper. If the individual-level or summarized data are available at a study level, then these can be incorporated into the analysis using hierarchical models, as has been previously proposed for the analysis of individual-level data [37]. This can take into account the heterogeneity between studies in a more principled way, particularly if some of the studies provide information on the genetic associations with both the risk factor and outcome.

### Weight of evidence from Mendelian randomization

In a hierarchy of evidence, Mendelian randomization investigations have been advocated as providing “critical evidence” on risk factor–outcome relationships [38]. However, the true weight of evidence in each case depends strongly on the plausibility of the instrumental variable assumptions for the genetic variants. If the function of the genetic variants is poorly understood, and there is little consistency in the causal effect estimates from multiple variants, then a causal conclusion is in doubt. A non-null Mendelian randomization estimate indicates that genetic predictors of the risk factor are also associated with the outcome, but there may be alternative causal pathways other than that through the risk factor of interest. This is particularly likely if a large number of variants are included in the analysis, and/or if the justification for using the variants in the analysis is solely on the basis of observational associations with the risk factor. Additionally, conclusions may still be limited by a lack of power, particularly if the genetic variants only explain a small proportion of the variance in the risk factor.

### Conclusion

In conclusion, we have here explained why Mendelian randomization is a useful approach for the assessment of risk factors as potential targets for clinical intervention. We have demonstrated how published data enable efficient analysis strategies for Mendelian randomization experiments. This is a timely development in view of the increasing public availability of genetic association estimates in large datasets. The efficiency of these analyses can be improved by using multiple variants in each gene region, but correlation between the variants must be accounted for.

## Electronic supplementary material

Below is the link to the electronic supplementary material.

Supplementary material 1 (pdf 149 KB)
