# Supplementary material for: Using published data in Mendelian randomization: a blueprint for efficient identification of causal risk factors
Source: Eur J Epidemiol. 2015 Mar 15;30(7):543–52. doi: 10.1007/s10654-015-0011-z (PMC4516908; doi:10.1007/s10654-015-0011-z)
Supplement: Supplementary file 1 — Supplementary material 1 (pdf 149 KB) [file 10654_2015_11_MOESM1_ESM.pdf]

Web Appendix

A.1 List of consortia with publicly-available GWAS data

We list consortia which have made genome-wide association data publicly available by March 2014 (Web Table A1). Some studies have released data only on the p-values and directions of effect of genetic variants, not the beta-coefficients. Unfortunately, such data cannot be used to estimate a causal effect parameter without making strong distributional assumptions to derive the relevant beta-coefficients and standard errors.

Further data resources can be found (subject to registration/approval) on the National Institute of Health (NIH) database of Genotypes and Phenotypes (dbGAP) (www.ncbi.nlm.nih.gov/gap), the European Genome–Phenome Archive (EGA) (https://www.ebi.ac.uk/ega/home), and GWAS Central (http://www.gwascentral.org/). Data from the Wellcome Trust Case-Control Consortium (WTCCC) consortium (http://www.wtccc.org.uk/) and the National Institute on Drug Abuse (NIDA) (https://nidagenetics.org/download_data.html) is also available to bona fide researchers on request subject to committee approval. Many relevant datasets for cancer outcomes can be found through the International Cancer Genome Consortium (ICGC) at http://docs.icgc.org/access-raw-data.

Consortium	Outcome	Website
AMDGC	age-related macular degeneration	http://www.sph.umich.edu/csg/abecasis/public/amdgene2012 [1]
APIDOGen	adiponectin	http://www.mcgill.ca/genepi/adipogen-consortium [2]
CARDIoGRAM-plusC4D	coronary artery disease	http://www.cardiogramplusc4d.org/downloads
CKDGen	chronic kidney disease	http://www.nhlbi.nih.gov/research/intramural/researchers/pi/fox-caroline/ckdgen-meta-analysis-data/datasets.html
DIAGRAM	type 2 diabetes	http://diagram-consortium.org/downloads.html
EGG	anthropometric traits in children	http://egg-consortium.org
GABRIEL	asthma	http://www.cng.fr/gabriel/results.html
GEFOS	osteoporosis	http://www.gefos.org/?q=content/data-release [1]
GIANT	anthropometric traits	http://www.broadinstitute.org/collaboration/giant/index.php/Data_Release [1]
GLGC	lipids	http://www.sph.umich.edu/csg/abecasis/public/lipids2013
ICBP	blood pressure and derived measures	http://www.ncbi.nlm.nih.gov/projects/gap/cgi-bin/study.cgi?study_id=phs000585.v1.p1
IIBDGC	inflammatory bowel disease, Crohn's disease and ulcerative colitis	http://www.ibdgenetics.org/downloads.html
ImmunoBase	immunologically related diseases	https://www.immunobase.org/downloads
MAGIC	glycaemic traits	http://www.magicinvestigators.org/downloads
Metabolomics	metabolites, xenobiotics and metabolite ratios	http://metabolomics.helmholtz-muenchen.de/gwas/index.php?task=download
PGC	ADHD, bipolar disorder, major depression and schizophrenia	https://pgc.unc.edu/Sharing.php
RAC	rheumatoid arthritis	http://plaza.umin.ac.jp/~yokada/datasource/software.htm
TAG	tobacco related measures	https://pgc.unc.edu/Sharing.php
T1DGC	type 1 diabetes	http://www.t1dbase.org/page/SNPDossierView

Web Table A1: Consortia with publicly-available data on genetic associations with outcomes.

Abbreviations: AMDGC = Age-related Macular Degeneration Genetics Consortium, CARDIoGRAMplusC4D = Coronary Artery Disease Genomewide Replication and Meta-analysis plus The Coronary Artery Disease Genetics, CKDGen = Chronic Kidney Disease Genetics, DIAGRAM = Diabetes Genetics Replication And Meta-analysis, EGG = Early Growth Genetics Consortium, GABRIEL = Genetic and Environmental Causes of Asthma in the European Community, GEFOS = Genetic Factors for Osteoporosis Consortium, GIANT = Genetic Investigation of Anthropometric Traits, GLGC = Global Lipids Genetics Consortium, ICBP = International Consortium for Blood Pressure, IIBDGC = International Inflammatory Bowel Disease Genetics Consortium, MAGIC = Meta-Analyses of Glucose and Insulin-related traits Consortium, PGC = Psychiatric Genomics Consortium, RAC = Rheumatoid Arthritis Consortium, TAG = Tobacco and Genetics Consortium, T1DGC = Type 1 Diabetes Genetics Consortium

[1]Only data on p-values and directions of association are currently available.

[2]Only data on p-values and directions of association are currently available. Beta-coefficients for selected variants are available on request.

A.2 R code for implementing an inverse-variance weighted Mendelian randomization analysis with uncorrelated genetic variants

Code for implementing an inverse-variance weighted Mendelian randomization analysis in the statistical software package R [1]:

```
x      = c(-0.026, -0.044, -0.038, -0.023, -0.018)    # genetic associations with risk factor
                                                      # for five genetic variants
sigmax = c( 0.004,  0.004,  0.004,  0.003,  0.004)    # standard errors
y      = c(-log(1.07), log(0.85), log(0.90), log(0.94), log(0.96))
               # genetic associations with outcome (log odds ratios)
sigmay = c((log(1.13)-log(1.01))/1.96/2, (log(0.90)-log(0.80))/1.96/2,
           (log(0.96)-log(0.85))/1.96/2, (log(0.99)-log(0.90))/1.96/2,
           (log(1.03)-log(0.89))/1.96/2)
               # standard errors

cat("Pooled estimate from inverse-variance weighted method: ",
    sum(x*y/sigmay^2)/sum(x^2/sigmay^2),
    " \nStandard error: ", sqrt(1/sum(x^2/sigmay^2)))
               # results

# Note: The data above are taken from reference [27]. The headline result in the paper's
#  abstract for a 30% reduction in LDL-c is obtained as exp(3.066309*log(0.7)) = 0.334983.
#  Hence an odds ratio of 0.33 and a risk reduction of 67%.
```

A.3 R code for implementing a likelihood-based two-sample Mendelian randomization analysis with uncorrelated genetic variants

Code for implementing an likelihood-based Mendelian randomization analysis in the statistical software package R. If the data are not from two non-overlapping samples, there will be correlation between the genetic associations with the risk factor and outcome which should be accounted for in the analysis model [2]. A web tool for implementing this method is provided at http://spark.rstudio.com/sb452/summarized/.

```
x      = c(-0.026, -0.044, -0.038, -0.023, -0.018)    # genetic associations with risk factor
                                                      # for five genetic variants
sigmax = c( 0.004,  0.004,  0.004,  0.003,  0.004)    # standard errors
y      = c(-log(1.07), log(0.85), log(0.90), log(0.94), log(0.96))
               # genetic associations with outcome (log odds ratios)
sigmay = c((log(1.13)-log(1.01))/1.96/2, (log(0.90)-log(0.80))/1.96/2,
           (log(0.96)-log(0.85))/1.96/2, (log(0.99)-log(0.90))/1.96/2,
           (log(1.03)-log(0.89))/1.96/2)
               # standard errors

loglikelihood <- function(param) {                    # log-likelihood function
  return(1/2*sum((x-param[1:length(x)])^2/sigmax^2)+1/2*
   sum((y-param[length(x)+1]*param[1:length(x)])^2/sigmay^2)) }

opt = optim(c(x, sum(x*y/sigmay^2)/sum(x^2/sigmay^2)),
            loglikelihood, hessian=TRUE, control = list(maxit=25000))
               # optimization command

cat("Pooled estimate from likelihood-based method: ", opt$par[length(x)+1],
    " \nStandard error: ", sqrt(solve(opt$hessian)[length(x)+1,length(x)+1]))
               # results

# Note: The data above are taken from reference [27]. The headline result in the paper's
#  abstract for a 30% reduction in LDL-c is obtained as exp(3.074619*log(0.7)) = 0.3339916.
#  Hence an odds ratio of 0.33 and a risk reduction of 67%.

cat("p-value for heterogeneity test: ", round(pchisq(2*opt$value, df=length(x)-1, lower.tail=FALSE),3))
```

A.4 R code for implementing a likelihood-based two-sample Mendelian randomization analysis with correlated genetic variants

Code for implementing an likelihood-based Mendelian randomization analysis with correlated genetic variants in the statistical software package R. For illustrative purposes, we assume a correlation of +0.2 between each pair of genetic variants (so $r^2 = 0.04$):

```
x      = c(-0.026, -0.044, -0.038, -0.023, -0.018)    # genetic associations with risk factor
                                                       # for five genetic variants
sigmax = c( 0.004,  0.004,  0.004,  0.003,  0.004)    # standard errors
y      = c(-log(1.07), log(0.85), log(0.90), log(0.94), log(0.96))
               # genetic associations with outcome (log odds ratios)
sigmay = c((log(1.13)-log(1.01))/1.96/2, (log(0.90)-log(0.80))/1.96/2,
           (log(0.96)-log(0.85))/1.96/2, (log(0.99)-log(0.90))/1.96/2,
           (log(1.03)-log(0.89))/1.96/2)
               # standard errors

rho = matrix(c(1, 0.2, 0.2, 0.2, 0.2,                 # correlation matrix
             0.2, 1,   0.2, 0.2, 0.2,
             0.2, 0.2, 1,   0.2, 0.2,
             0.2, 0.2, 0.2, 1,   0.2,
             0.2, 0.2, 0.2, 0.2, 1), nrow=length(x), ncol=length(x))

Sigmax = array(NA, dim=c(length(x),length(x)));
Sigmay = array(NA, dim=c(length(x),length(x)));

for (k1 in 1:length(x)) {
 for (k2 in 1:length(x)) {
   Sigmax[k1,k2] = sigmax[k1]*sigmax[k2]*rho[k1,k2]
   Sigmay[k1,k2] = sigmay[k1]*sigmay[k2]*rho[k1,k2]
   }
 }
Taux = solve(Sigmax); Tauy = solve(Sigmay)

loglikelihoodcorrel <- function(param) {               # log-likelihood function
   return(1/2*t(x-param[1:length(x)])%*%Taux%*%(x-param[1:length(x)])+1/2*
     t(y-param[length(x)+1]*param[1:length(x)])%*%Tauy%*%
      (y-param[length(x)+1]*param[1:length(x)])) }

opt = optim(c(x, sum(x*y/sigmay^2)/sum(x^2/sigmay^2)),
            loglikelihoodcorrel, hessian=TRUE, control = list(maxit=25000))
               # optimization command

cat("Pooled estimate from likelihood-based method: ", opt$par[length(x)+1],
    " \nStandard error: ", sqrt(solve(opt$hessian)[length(x)+1,length(x)+1]))
               # results

cat("p-value for heterogeneity test: ", round(pchisq(2*opt$value, df=length(x)-1, lower.tail=FALSE),3))
```

A.5 WinBUGS code for implementing a likelihood-based two-sample Mendelian randomization analysis with uncorrelated genetic variants

Code for implementing an likelihood-based Mendelian randomization analysis in the statistical software package WinBUGS [3]. The WinBUGS code is recommended for use when there are large (more than 10) numbers of variants, and as at least a sensitively analysis with fewer variants.

```
model {
 beta1 ~ dnorm(0, 0.000001)       # prior for causal effect
  for (k in 1:K) {                # loop over K genetic variants
    xi[k] ~ dnorm(0, 0.000001)    # prior for mean of G-X association
    taux[k] <- pow(sigmax[k], -2) # precision of G-X association
    tauy[k] <- pow(sigmay[k], -2) # precision of G-Y association
    x[k] ~ dnorm(xi[k], taux[k])  # normal distribution of G-X association
    eta[k] <- beta1*xi[k]         # mean of G-Y association
    y[k] ~ dnorm(eta[k], tauy[k]) # normal distribution of G-Y association
    }
 }
```

The WinBUGS programme can be called in R using the following code:

```
library(R2WinBUGS)

x      = c(-0.026, -0.044, -0.038, -0.023, -0.018)    # genetic associations with risk factor
                                                      # for five genetic variants
sigmax = c( 0.004,  0.004,  0.004,  0.003,  0.004)    # standard errors
y      = c(-log(1.07), log(0.85), log(0.90), log(0.94), log(0.96))
               # genetic associations with outcome (log odds ratios)
sigmay = c((log(1.13)-log(1.01))/1.96/2, (log(0.90)-log(0.80))/1.96/2,
           (log(0.96)-log(0.85))/1.96/2, (log(0.99)-log(0.90))/1.96/2,
           (log(1.03)-log(0.89))/1.96/2)
               # standard errors
K = 5

data <- list ("x", "y", "sigmax", "sigmay", "K")
inits <- function() {list (beta1=0)}
parameters <- c("beta1")

summarized.sim <- bugs (data, inits, parameters, "C:/foo/model.txt",
 n.chains=1, n.burnin=1000, n.iter=11000, n.sims=10000, bugs.directory="C:/foo/WinBUGS14/")

cat("Pooled estimate from likelihood-based method: ", summarized.sim$summary[1,1],
    " \n95% credible interval: ", summarized.sim$summary[1,3], ", ", summarized.sim$summary[1,7])
```

A.6 WinBUGS code for implementing a likelihood-based two-sample Mendelian randomization analysis with correlated genetic variants

Code for implementing an likelihood-based Mendelian randomization analysis in the statistical software package WinBUGS assuming the genetic variants are correlated in their distribution (that is, they are in linkage disequilibrium).

```
model {
 beta1 ~ dnorm(0, 0.000001)                   # prior for causal effect
 Tau0[1:K, 1:K] <- inverse(Sigma0[1:K, 1:K]) # variance-covariance matrix of prior
                                              # for mean of G-X association
 xi[1:K]  ~ dmnorm(mu0[1:K], Tau0[1:K, 1:K]) # prior for mean of G-X association
for (k in 1:K) { eta[k] <- beta1*xi[k] }     # mean of G-Y association
 x[1:K] ~ dmnorm(xi[1:K],  Taux[1:K, 1:K])   # multivariate normal distribution of G-X associations
 y[1:K] ~ dmnorm(eta[1:K], Tauy[1:K, 1:K])   # multivariate normal distribution of G-Y associations
 }
```

The WinBUGS programme can be called in R using the following code, which for illustrative purposes assumes a correlation of +0.2 between each pair of genetic variants:

```
library(R2WinBUGS)

x      = c(-0.026, -0.044, -0.038, -0.023, -0.018)    # genetic associations with risk factor
                                                       # for five genetic variants
sigmax = c( 0.004,  0.004,  0.004,  0.003,  0.004)    # standard errors
y      = c(-log(1.07), log(0.85), log(0.90), log(0.94), log(0.96))
               # genetic associations with outcome (log odds ratios)
sigmay = c((log(1.13)-log(1.01))/1.96/2, (log(0.90)-log(0.80))/1.96/2,
           (log(0.96)-log(0.85))/1.96/2, (log(0.99)-log(0.90))/1.96/2,
           (log(1.03)-log(0.89))/1.96/2)
               # standard errors
K = 5; Sigma0 = diag(1, K); mu0 = rep(0, K)

rho = matrix(c(1, 0.2, 0.2, 0.2, 0.2,
             0.2, 1,   0.2, 0.2, 0.2,
             0.2, 0.2, 1,   0.2, 0.2,
             0.2, 0.2, 0.2, 1,   0.2,
             0.2, 0.2, 0.2, 0.2, 1), nrow=K, ncol=K)

Sigmax = array(NA, dim=c(K,K)); Sigmay = array(NA, dim=c(K,K));

for (k1 in 1:K) {
 for (k2 in 1:K) {
   Sigmax[k1,k2] = sigmax[k1]*sigmax[k2]*rho[k1,k2]
   Sigmay[k1,k2] = sigmay[k1]*sigmay[k2]*rho[k1,k2]
   }
 }
Taux = solve(Sigmax); Tauy = solve(Sigmay)

data <- list ("x", "y", "Taux", "Tauy", "K", "Sigma0", "mu0")
inits <- function() {list (beta1=0)}
parameters <- c("beta1")
summarized.sim <- bugs (data, inits, parameters, "C:/foo/model.txt",
 n.chains=1, n.burnin=1000, n.iter=11000, n.sims=10000, bugs.directory="C:/foo/WinBUGS14/")

cat("Pooled estimate from likelihood-based method: ", summarized.sim$summary[1,1],
    " \n95% credible interval: ", summarized.sim$summary[1,3], ", ", summarized.sim$summary[1,7])
```

A.7 Supplementary methods for applied example

Genetic variants

Details of the genetic variants used in the applied example of the paper are given in Web Table A2 (genetic variants in and around the *CASR* gene region) and Web Table A3 (genetic variants in all gene regions taken from O'Seaghdha et al. [4]). In the EPIC-InterAct study, all variants were extracted for genotyping on the Illumina 660W-Quad Bead Chip (Illumina, San Diego, CA, USA) or Illumina Cardio-Metabochip (Illumina, San Diego, CA, USA). For each variant, we give the chromosome and gene region in which the variant is located, the effect and other alleles, and the coefficients for the genetic associations with calcium levels and fasting glucose together with corresponding p-values. The associations with calcium levels are estimated in the EPIC-InterAct dataset using linear regression with adjustment for age, sex, and centre. The associations with fasting glucose are publicly-available data contributed by MAGIC investigators and have been downloaded from www.magicinvestigators.org. Associations with covariates in Figure 2 are also calculated in the EPIC-InterAct using linear regression with adjustment for age, sex, and centre. A slightly larger sample size (up to 7311 individuals) was available for some of the covariates, depending on the availability of data on the covariate in question (minimum sample size was 5218).

All genetic variants on the Cardio-Metabochip located within a 500kb range of the *CASR* gene region were considered for the analysis of genetic variants in and around the *CASR* gene region. Proxies for the variants listed by O'Seaghdha et al. [4]) on the Cardio-Metabochip used for the analysis of genetic variants in different gene regions were found using the SNP Annotation and Proxy Search (SNAP, http://www.broadinstitute.org/mpg/snap/ldsearch.php) [5]. Partial F statistics for the variants in and around the *CASR* gene region were estimated in an analysis of variance (ANOVA) as 3.4 with 17 variants (on 17, 6314 degrees of freedom [df]), 7.9 with 6 variants (on 6, 6325 df), and 30.6 with the lead variant only (on 1, 6330 df).

Genetic associations with covariates

P-values for the associations of covariates with each of the genetic variants in turn are given in Web Table A4 for variants in and around the *CASR* gene region, and in Web Table A5 for variants in all gene regions. We see that p-values for variants in and around the *CASR* gene region are not more extreme than would be expected by chance (3 of $17 \times 13 = 221$ p-values less than 0.05, minimum p-value 0.034). In contrast, p-values for variants in different gene regions were more extreme than would be expected by chance (16 of $10 \times 13 = 130$ p-values less than 0.05, minimum p-value 1×10^{-13}). In particular, rs780093 was associated with a large number of covariates. If this variant is omitted from the analysis using variants from different gene regions, the causal effect estimate is dominated by the variant from the *CASR* gene region.

Instrumental variable analysis methods

The likelihood-based analysis using variants from the *CASR* gene region was performed using the likelihood-based method with correlated variants in WinBUGS [6]. The matrix for the correlations between genetic variants was estimated in the EPIC-InterAct dataset. If the individual-level data for estimating correlations were not available, they could be estimated from the literature. A web interface for obtaining such correlations is the SNP Annotation and Proxy Search (SNAP; http://www.broadinstitute.org/mpg/snap/ldsearchpw.php). Normal priors with mean 0 and variance 1000^2 were used for the parameters. The causal estimate was taken as the posterior mean, and the 95% credible interval as the 2.5th to the 97.5th percentile of the posterior distribution.

The posterior distributions of the causal effect parameter β_L from the Bayesian likelihood-based analyses using 17, 6, and 1 genetic variant are displayed in Web Figure A1. The posterior distributions are asymmetric, with longer tails in the positive than in the negative direction.

Assessing population stratification

To assess population stratification, we calculated the minor allele frequencies of the 17 variants in and around the *CASR* gene region in each of the 21 centres in EPIC-InterAct which provided data to the analysis. A meta-analysis was performed to assess whether the minor allele frequencies were similar in each centre. Additionally, the latitude of each centre was recorded by searching for the toponym in the centre name in Google Maps (www.google.com/maps) and using the lab feature "LatLng Marker". A meta-regression was performed on the latitude variable to assess whether differences in the minor allele frequencies between centres were driven by geographic location. Web Table A6 reports the I^2 measure of the degree of heterogeneity in the minor allele frequencies, together with a 95% confidence interval for I^2 and p-value for Cochrane's Q statistic, and the amount of heterogeneity accounted for by the latitude variable in the meta-regression model (R^2 statistic), together with the p-value for the latitude coefficient in the model. We conclude that there is substantial heterogeneity in the minor allele frequencies between the centres in the EPIC-InterAct dataset, and that the latitude of the centres explains a large proportion of this heterogeneity for several variants. Although this heterogeneity between centres will not lead to bias, it may indicate a latent substructure in the EPIC-InterAct and MAGIC populations not accounted for by the division into centres/studies, which in turn may lead to bias in the Mendelian randomization estimates.

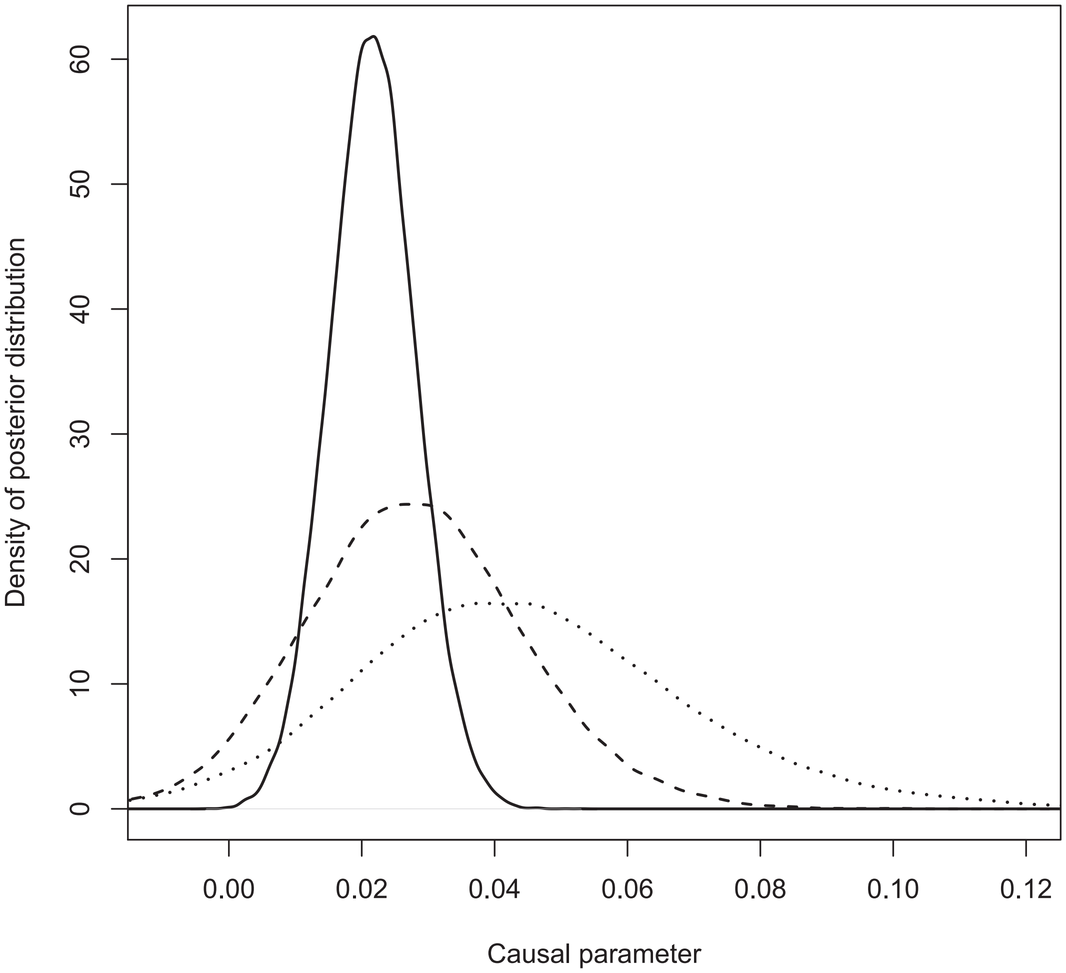

Web Figure A1: Posterior distributions of causal effect parameter β_L from likelihood-based analysis method in a Bayesian framework using 17 (solid line), 6 (dashed line), and 1 genetic variant (dotted line) obtained from Monte Carlo Markov chain (MCMC) sampling.

Genetic variant	Chr	Gene region	Effect allele	Other allele	Calcium levels (mmol/L)	p	Fasting glucose (mM)	p
rs9824133	3	IQCB1	C	T	0.0062	0.007	0.0030	0.15
rs7648041	3	CASR	T	C	0.0015	0.55	-0.0001	0.96
rs1354162	3	CASR	T	G	-0.0059	0.081	-0.0004	0.92
rs1801725	3	CASR	T	G	0.0182	< 0.001	0.0057	0.058
rs17199211	3	CASR–CSTA	T	C	0.0001	0.98	0.0006	0.79
rs4247197	3	CASR–CSTA	G	A	-0.0056	0.21	-0.0008	0.82
rs12491981	3	CSTA	C	T	-0.0050	0.010	0.0017	0.64
rs6798997	3	CSTA–CCDC58	A	G	-0.0060	0.18	-0.0011	0.60
rs13081171	3	CCDC58	G	T	-0.0033	0.72	-0.0002	0.94
rs7647266	3	FAM162A	T	C	-0.0027	0.24	0.0001	0.95
rs1127343	3	FAM162A	G	A	0.0083	< 0.001	-0.0006	0.79
rs3762637	3	KPNA1	T	C	-0.0026	0.44	-0.0015	0.60
rs12496410	3	KPNA1	C	A	-0.0065	0.065	-0.0012	0.71
rs9811581	3	PARP9	C	T	-0.0018	0.42	-0.0017	0.44
rs7611002	3	PARP15–PARP14	A	C	0.0012	0.73	-0.0032	0.27
rs10433340	3	PARP14	A	G	0.0002	0.93	-0.0012	0.59
rs2063515	3	PARP14	A	G	-0.0013	0.64	0.0025	0.50

Web Table A2: Genetic variants in and around the calcium-sensing receptor (*CASR*) gene region: chromosome (Chr) and gene regions, effect and other alleles, per allele associations with calcium levels (mmol/L) and fasting glucose (mM) with corresponding p-values.

Genetic variant	Available proxy	Chr	Gene region	Effect allele	Other allele	Calcium levels (mmol/L)	p	Fasting glucose (mM)	p
rs780093	rs780093	2	GCKR	T	C	0.0070	0.002	-0.0280	2×10^{-38}
rs838705	rs838709	2	DGKD	C	A	0.0033	0.19	-0.0023	0.29
rs9831754	rs1355625	3	intergenic	A	G	0.0008	0.73	0.0056	0.008
rs17251221	rs1801725	3	CASR	T	G	0.0182	< 0.001	0.0057	0.058
rs13195786	rs4712380	6	intergenic	T	G	0.0009	0.72	-0.0009	0.72
rs682238	rs1949365	6	intergenic	A	G	-0.0030	0.37	0.0001	0.98
rs1780159	rs12003180	9	intergenic	T	C	-0.0121	0.029	-0.0008	0.88
rs3847646	rs377765	11	intergenic	T	C	-0.0001	0.97	-0.0021	0.33
rs12150338	rs12051752	17	WDR81	T	G	0.0007	0.79	-0.0031	0.18
rs6091737	rs6022755	20	intergenic	A	C	0.0042	0.075	-0.0013	0.55

Web Table A3: Genetic variants in different gene regions: genetic variant from O'Seaghdha et al. [4] and proxy on Cardio-Metabochip, chromosome (Chr) and gene regions, effect and other alleles, per allele associations with calcium levels (mmol/L) and fasting glucose (mM) with corresponding p-values.

Covariate	rs9824133	rs7648041	rs1354162	rs1801725	rs17199211	rs4247197	rs12491981	rs6798997	rs13081171	rs7647266	rs1127343	rs3762637	rs12496410	rs9811581	rs7611002	rs10433340	rs2063515
BMI	0.24	0.47	0.35	0.64	0.62	0.90	0.76	0.71	0.97	0.65	0.89	0.58	0.29	0.74	0.69	0.13	0.07
SBP	0.27	0.35	0.66	0.63	0.05	0.78	0.46	0.25	0.82	0.14	0.37	0.78	0.28	0.14	0.38	0.64	0.15
DBP	0.36	0.32	0.46	0.98	0.14	0.39	0.39	0.52	0.37	0.11	0.48	0.75	0.44	0.24	0.16	0.70	0.84
Total chol	1.00	0.90	0.74	0.41	0.86	0.46	0.58	0.28	0.25	0.92	0.31	0.79	**0.04**	0.58	0.87	0.95	0.23
HDL-c	0.39	0.97	0.31	0.51	0.97	0.46	0.46	0.61	0.51	0.14	0.45	0.96	0.10	0.17	0.65	0.74	0.20
Non-HDL-c	0.80	0.92	1.00	0.31	0.82	0.65	0.79	0.36	0.19	0.71	0.21	0.77	0.16	0.92	0.99	0.96	0.11
log Trig	0.27	0.74	0.88	0.15	0.58	0.88	0.91	0.43	0.23	0.56	0.10	0.63	0.26	0.37	0.98	1.00	0.83
Apo A1	0.54	0.65	0.19	0.60	0.96	0.57	0.51	0.66	0.80	0.37	0.42	0.86	0.11	0.20	0.97	0.40	0.31
Apo B	0.33	0.87	0.88	0.55	0.52	0.91	0.92	0.43	0.36	0.83	0.56	0.98	0.39	0.86	0.84	0.65	**0.05**
log CRP	0.97	0.20	0.70	0.77	0.59	0.72	0.69	0.60	0.62	0.92	0.29	0.70	0.61	0.64	0.65	0.55	0.86
Albumin	0.12	0.87	0.86	0.73	0.84	0.56	0.41	0.35	0.77	0.76	0.48	0.26	0.65	0.51	0.60	0.51	0.74
Creatin	0.99	0.57	0.14	0.18	0.71	0.11	0.13	0.23	0.35	0.72	0.61	0.28	0.36	0.50	0.10	0.96	0.75
Uric acid	0.53	0.44	0.79	0.37	0.63	0.88	0.79	0.55	0.52	0.89	0.11	0.57	0.35	0.37	**0.03**	0.95	0.30

Web Table A4: P-values for associations of covariates with all genetic variants in and around the *CASR* gene region. P-values less than 0.05 are shown in bold face.

Covariate	rs780093	rs838709	rs1355625	rs1801725	rs4712380	rs1949365	rs12003180	rs377765	rs12051752	rs6022755
Body mass index	0.14	0.74	0.11	0.64	0.17	0.56	0.46	0.34	0.22	0.28
Systolic blood pressure	0.56	0.95	0.43	0.63	0.89	0.90	0.94	0.13	0.20	0.18
Diastolic blood pressure	0.78	0.46	0.15	0.98	0.84	0.51	0.81	0.23	0.76	0.09
Total cholesterol	$<$ **0.001**	0.98	0.27	0.41	0.39	0.32	0.90	0.44	**0.02**	0.45
HDL-cholesterol	**0.02**	0.70	0.37	0.51	0.13	0.81	0.08	0.49	0.11	0.48
Non-HDL-c	$<$ **0.001**	0.93	0.16	0.31	0.18	0.30	0.47	0.31	0.09	0.31
log Triglycerides	$<$ **0.001**	0.87	0.50	0.15	0.15	**0.01**	0.38	0.73	0.15	0.15
Apolipoprotein A1	0.67	0.72	0.55	0.60	0.19	0.59	0.14	0.46	0.15	0.98
Apolipoprotein B	$<$ **0.001**	0.86	0.15	0.55	0.23	0.48	0.86	0.19	0.15	0.15
log C-reactive protein	$<$ **0.001**	**0.04**	**0.03**	0.77	0.58	0.54	0.95	0.53	0.69	**0.02**
Albumin	**0.05**	0.64	0.91	0.73	0.50	0.25	0.49	0.50	0.86	0.32
Creatin	$<$ **0.001**	0.39	0.45	0.18	**0.02**	0.23	0.91	0.53	0.58	**0.03**
Uric acid	$<$ **0.001**	0.34	0.32	0.37	0.38	0.39	0.67	0.18	0.17	0.82

Web Table A5: P-values for associations of covariates with genetic variants in different gene regions. P-values less than 0.05 are shown in bold face.

Genetic variant	Heterogeneity		Meta-regression	
	I^2 (95% CI)	p-value	R^2	p-value
rs9824133	86.1 (80.1, 90.3)	***	67.6	***
rs7648041	33.0 (0.0, 60.5)		44.7	*
rs1354162	85.6 (79.3, 90.0)	***	100.0	***
rs1801725	83.6 (76.1, 88.8)	***	45.3	**
rs17199211	60.5 (36.4, 75.5)	***	17.0	
rs4247197	79.0 (68.4, 86.0)	***	91.4	***
rs12491981	76.4 (64.2, 84.5)	***	98.8	***
rs6798997	86.8 (81.2, 90.7)	***	58.8	***
rs13081171	48.8 (15.1, 69.1)	**	0.0	
rs7647266	43.7 (5.7, 66.4)	*	0.0	
rs1127343	44.5 (7.3, 66.8)	*	0.0	
rs3762637	87.9 (82.9, 91.4)	***	66.7	***
rs12496410	79.5 (69.3, 86.3)	***	42.1	**
rs9811581	54.9 (26.3, 72.4)	**	1.6	
rs7611002	21.5 (0.0, 53.9)		0.0	
rs10433340	81.7 (73.0, 87.6)	***	68.3	***
rs2063515	73.1 (58.6, 82.6)	***	77.7	***

Web Table A6: Heterogeneity in minor allele frequencies between 21 centres of EPIC-InterAct for genetic variants in and around the *CASR* gene region: I^2 statistic (%) with 95% confidence interval (CI) and p-value for Q statistic; meta-regression of minor allele frequencies on latitude variable: proportion of heterogeneity explained (R^2 statistic, %) and p-value of coefficient

p-values: * = < 0.05, ** = < 0.01, *** = < 0.001

References

[1] R Development Core Team. *R: A language and environment for statistical computing*. R Foundation for Statistical Computing, Vienna, Austria 2011. URL http://www.R-project.org.

[2] Burgess S, Butterworth A, Thompson S. Mendelian randomization analysis with multiple genetic variants using summarized data. *Genetic Epidemiology* 2013; **37**(7):658–665, doi:10.1002/gepi.21758.

[3] Spiegelhalter D, Thomas A, Best N, Lunn D. WinBUGS version 1.4 user manual. *Technical Report*, MRC Biostatistics Unit, Cambridge, UK 2003.

[4] O'Seaghdha CM, Yang Q, Glazer NL, Leak TS, Dehghan A, Smith AV, Kao WL, Lohman K, Hwang SJ, Johnson AD, *et al.*. Common variants in the calcium-sensing receptor gene are associated with total serum calcium levels. *Human Molecular Genetics* 2010; **19**(21):4296–4303, doi:10.1093/hmg/ddq342.

[5] Johnson A, Handsaker R, Pulit S, Nizzari M, O'Donnell C, de Bakker P. SNAP: a web-based tool for identification and annotation of proxy SNPs using HapMap. *Bioinformatics* 2008; **24**(24):2938–2939, doi:10.1093/bioinformatics/btn564.

[6] Lunn D, Thomas A, Best N, Spiegelhalter D. WinBUGS – A Bayesian modelling framework: Concepts, structure, and extensibility. *Statistics and Computing* 2000; **10**(4):325–337, doi:10.1023/A:1008929526011.
